# Investigation of the Effect of Preconditioning with Ultrasound on Fat Graft Survival

**DOI:** 10.1007/s00266-025-04771-6

**Published:** 2025-03-20

**Authors:** Arif Yılmaz, Bilge Kağan Yılmaz, Çiğdem Karaca, Necmettin Karasu

**Affiliations:** 1 Department of Plastic Reconstructive and Aesthetic Surgery, Afyonkarahisar Health Science University, Afyonkarahisar, Turkey; 2 Department of Orthopaedic and Traumatology, Afyonkarahisar Health Science University, Afyonkarahisar, Turkey; 3 Department of Histology and Embryology, Gaziantep Islamic Science and Technology University, Gaziantep, Turkey; 4 Private Parkhayat Afyonkarahisar Hospital Department of Plastic Reconstructive and Aesthetic Surgery, Afyonkarahisar, Turkey

**Keywords:** Therapeutic ultrasound, Fat grafts, Vascularity

## Abstract

**Background:**

The use of fat grafts in plastic, reconstructive, and aesthetic surgery has been increasing. Although fat grafting has many advantages, there is limited evidence regarding its survival rates. Current studies focus on improving fat graft survival rates. This study aimed to evaluate the effect of therapeutic ultrasound (TERUS) application on fat graft survival.

**Methods:**

The study involved 42 adult male Wistar Albino rats, which were randomly divided into seven groups of six rats each: Group 1 served as the control group and received only fat grafting; Group 2 received preoperative daily TERUS for one week, followed by fat grafting; Group 3 underwent preoperative daily massage for one week, followed by fat grafting; Group 4 received preoperative daily TERUS for one week, followed by fat grafting and postoperative daily TERUS for one week; Group 5 underwent preoperative daily massage for one week, followed by fat grafting and postoperative daily massage for one week; Group 6 received postoperative daily TERUS for one week; and Group 7 received postoperative daily massage for one week. After volume and weight measurements, immunohistochemical evaluation was conducted using perilipin and PECAM-1. Apoptosis was assessed using the TUNEL method.

**Results:**

No statistically significant differences were observed in the macroscopic measurements. While TERUS increased the vascularization of fat grafts, it did not improve survival rates. The mean fat graft survival rate in the preoperative–postoperative massage group (Group 5) was statistically significantly higher than in the other groups.

**Conclusions:**

The findings of this study suggest that the massage effect of TERUS, independent of ultrasonic energy, may be beneficial for fat graft survival. Although TERUS increases the vascularity of fat grafts, it does not improve fat graft survival rates.

**No Level Assigned:**

This journal requires that authors assign a level of evidence to each submission to which Evidence-Based Medicine rankings are applicable. This excludes Review Articles, Book Reviews, and manuscripts that concern Basic Science, Animal Studies, Cadaver Studies, and Experimental Studies. For a full description of these Evidence-Based Medicine ratings, please refer to the Table of Contents or the online Instructions to Authors www.springer.com/00266.

## Introduction

Fat grafting is a widely utilized technique in plastic, aesthetic, and reconstructive surgery and has seen increasing popularity in recent years. It has various indications, including volume restoration for facial depressions and age-related changes for aesthetic purposes, volume augmentation in breast reconstruction following cancer surgery, and scar tissue regeneration due to its stem cell content [1].

The viability of fat grafts is highly variable across applications. Since the first use of fat grafting in the 1890s, there has been growing interest in improving graft viability through advancements in technology and medicine [2]. Many methods have been explored, including the use of endogenous substances such as platelet-rich plasma, growth factors (e.g., epidermal growth factor) and vascular endothelial growth factor, exogenous substances (e.g., botulinum toxin), and preconditioning of the recipient field; however, no universally accepted consensus has yet been established [3–6].

Preconditioning refers to any procedure performed to increase the success of an operation and is commonly employed in flap surgeries. It can involve botulinum toxin injection, invasive methods such as microporation, or non-invasive approaches such as therapeutic ultrasound (TERUS) [4, 6–9]. TERUS is a device used in physical therapy and rehabilitation to provide deep tissue heating. Studies have demonstrated that TERUS decreases inflammation and increases blood flow in the treated area. By increasing cell membrane permeability,

TERUS facilitates the diffusion of extracellular calcium ions into cells [10]. In addition, TERUS can decrease fat tissue and tighten skin by stimulating collagen synthesis when used at variable intensities [11–13]. It has also been employed in various animal studies focusing on tendon healing, scar healing, and enhancing the viability of skin flaps [9, 14, 15].

This study aimed to investigate the effect of TERUS on fat graft viability, considering its potential to reduce inflammation and increase vascularity.

## Materials and Methods

### Animals

This study was conducted with the approval of Afyon Kocatepe. The experiment complied with European Union Directive 2010/63/EU for the Protection of Experimental Animals and the regulations of the Afyon Kocatepe University Experimental Animals Ethics Committee. All applicable institutional and/or national guidelines for the care and use of animals were followed.

The study was designed as a single-blind randomized trial. Forty-two adult male Wistar Albino rats, with an average weight of 256.9 ± 54.4 g, were used in the study. Animals weighing less than 200 g and more than 500 g were excluded. The animals were housed individually in standard cages under controlled environmental conditions and provided with free access to standard pellet food and water.

## Experimental Groups

The animals were randomly divided into seven groups, with six rats in each group:

*Group 1*: Control (only fat grafting).

*Group 2*: Daily TERUS application with the device on for one week prior to surgery, followed by fat grafting.

*Group 3*: Daily massage using the TERUS probe (device off) for one week prior to fat grafting.

*Group 4*: Daily TERUS application for one week preoperatively, followed by fat grafting and an additional week of daily TERUS postoperatively.

*Group 5*: Daily massage using the TERUS probe (device off) for one week preoperatively, followed by fat grafting and an additional week of daily massage postoperatively.

*Group 6*: Daily TERUS application with the device on for one week postoperatively after fat grafting.

*Group 7*: Daily massage using the TERUS probe (device off) for one week postoperatively after fat grafting.

In Groups 3, 5, and 7, the handpiece of the TERUS device was used to evaluate the massage effect. For standardization purposes, daily massage was performed with the TERUS device turned off. These groups were formed with the aim of assessing the massage effect of the ultrasound probe.

For all groups, the donor area was the right inguinal region, and the recipient site was the subcutaneous tissue of the parascapular region.

## Surgical Procedures

**Fig. 1:**
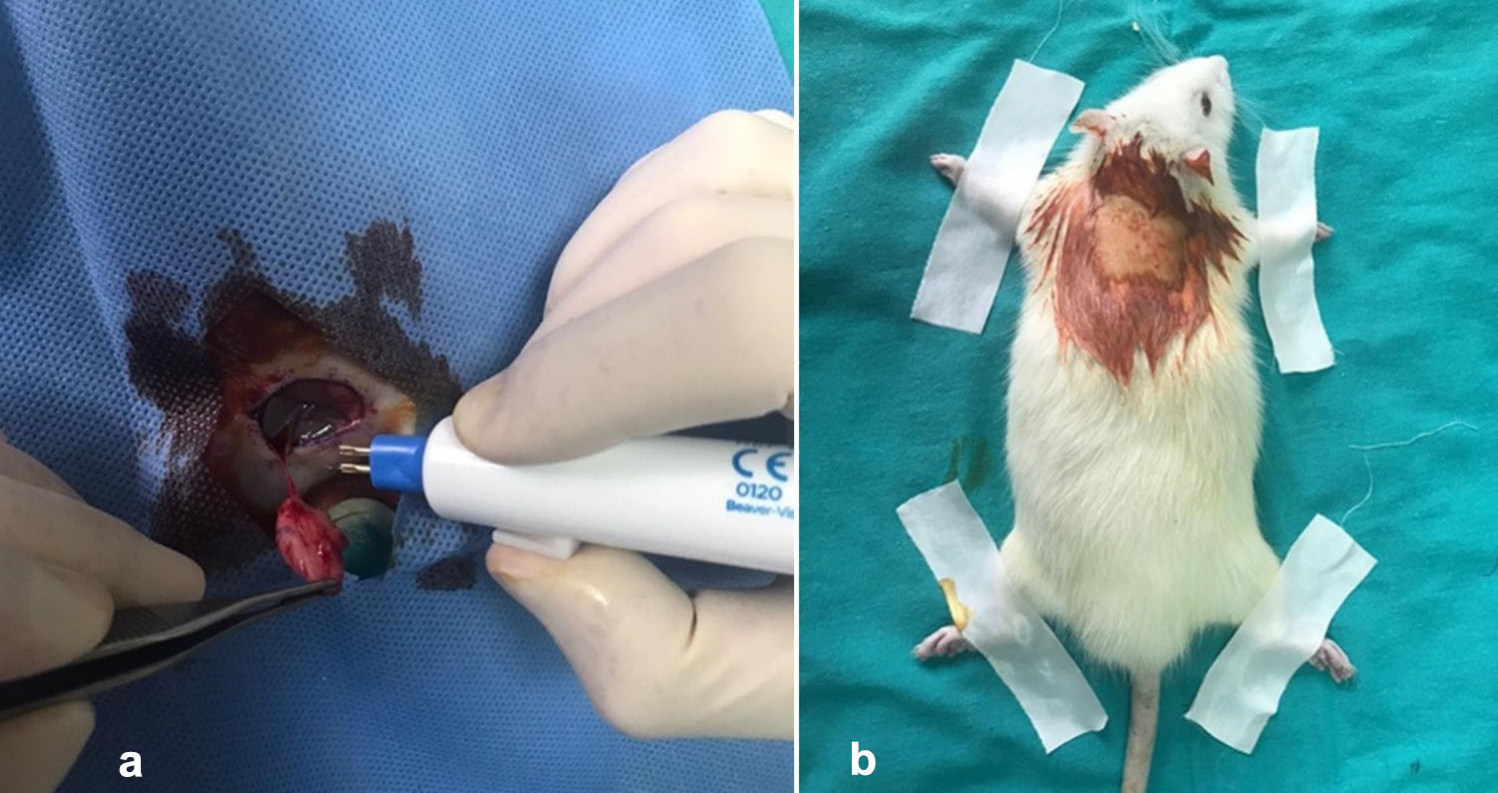
**a**. Inguinal fat graft excision, **b**. fat graft implantation area in the parascapular region.

The surgical procedures were performed as described by Temiz et al. [16]. After shaving the hair in the surgical area, disinfecting with povidone iodine (Batikon-Genesis Ilac A.S.), and covering the site with sterile blue scrubs, the rats were secured with tape. Under general anesthesia (50 mg/kg ketamine (Ketalar-Pfizer Inc.) and 5 mg/kg xylazine (Rompun® 2%, Bayer Türk Kimya Sanayi Ltd. Sti., Istanbul, Turkey), fat grafts were harvested from the right inguinal fat pad and implanted into the subcutaneous tissue of the parascapular region, superficial to the muscle fascia. The rats were positioned supine, with their extremities fixed. A parallel incision was made along the inguinal ligament, and blunt dissection was used to expose the femoral peduncle. The fat graft was dissected from the surrounding tissues (Fig. 1a). The epigastric artery and vein were identified and cauterized to isolate the fat tissue. The weight of the graft was measured, and its volume was calculated using Archimedes’ principle. The grafts were kept in wet gauze. The incision in the inguinal region was closed with absorbable sutures.

The rats were then positioned prone, and their extremities were fixed to prepare the recipient region. After disinfection, a 1.5-cm skin incision was made in the parascapular region, and sufficient subcutaneous tissue was dissected to insert a 1.5x1.5-cm sterile silicon layer (Fig. 1b). The silicone layer was used to ensure equal and standardized subcutaneous dissection in all subjects. After removing the silicone layer, the fat graft was inserted into the prepared subcutaneous pouch, and the skin incision was closed. The rats were returned to their cages for rest and recovery.

At the end of the eight-week follow-up period, general anesthesia was administered again. The rats were placed in the prone position, and the dorsal interscapular region was shaved. After disinfecting the site with povidone iodine, a 1.5-cm skin incision was made in the parascapular region, and the fat grafts were excised. Following the completion of all surgical procedures, the rats were sacrificed in accordance with ethical regulations.

## TERUS Application

Before applying TERUS, the size and shape of the ultrasound handpiece were traced onto plaster, which was then was cut to match the shape. TERUS was applied to the recipient area of each rat using the BTL-4000 device (Smart & Premium, BTL Türkiye, Ankara, Turkey). The dorsum of the animals was shaved to match the shape of the cut plaster, and the area where the ultrasound probe would be applied was marked with a pen. TERUS was applied to the marked dorsal region in a circular motion without exerting pressure on the skin. The application was performed with an intensity of 1 W/cm^2^, a carrier frequency of 3 MHz, and a duty factor of 100%. In the massage groups, the massage effect was investigated using the same circular motions but with the device turned off.

## Histopathological Analysis

The fat tissues obtained were fixed in 10% neutral formalin for four days. After fixation, the tissues were labeled and washed under running water for one day. The samples were subsequently kept in 70%, 80%, 90%, and pure alcohol solutions in sequence. Next, they were immersed in a xylene + alcohol mixture for 45 minutes, followed by two separate immersions in xylene for 45 minutes each. The samples were then infiltrated with liquid paraffin for one hour and embedded in paraffin blocks. From these blocks, 5-micron-thick slices were sectioned. The slices were mounted onto ordinary slides for routine examination and onto slides coated with polylysine for immunohistochemical examination. The slices on ordinary slides were stained with hematoxylin–eosin (HE) after deparaffinization. Slides with polylysine were stained with perilipin (bs-3789R 1/50), PECAM-1 (H-3) (sc-376764, 1/100) antibodies after deparaffinization. The slides were examined under a light microscope.

To evaluate apoptosis, additional 5-micron-thick slices were mounted onto polylysine slides and stained using the TUNEL method, following the protocol of the ApopTag® Plus Peroxidase In Situ Apoptosis Kit (S7101). These slides were also examined under a light microscope.

The slices stained with HE were scored histopathologically. Cyst formation, inflammation, and fibrosis were evaluated under a light microscope at 10x magnification, and a score was assigned based on the severity of the changes observed: 0 = no change, 1 = minimal, 2 = minimal to moderate, 3 = moderate, and 4 = moderate to severe. The data were subsequently analyzed statistically.

For image analysis, the Nikon NIS 4.2 Image Analysis Software was used. The HE-stained samples were examined at 10x magnification using a Nikon E-60 light microscope. An H-score was used to evaluate the immunohistochemically stained samples. Fat cells were counted in every sample under 20x magnification, with a total of 500 fat cells counted per sample. The cells were categorized as +1, +2, or +3 based on staining intensity, while unstained cells were assigned a score of 0.

The H-score was calculated using the formula: H-score = (I + 1) × number of stained cells [17].

The weights and volumes of the rats and fat grafts were measured and statistically analyzed according to the formulas provided below.

$${\text{Percentage of fat graft survival}} = \frac{{{\text{Volume of fat graft excised at week eighth}}}}{{{\text{volume of fat graft at the beginning}}}} \times {\text{100}}.$$

$$\frac{{{\text{Volume}}}}{{{\text{Weight}}}}{\text{change}\, \text{percentage}} = \frac{{\left( {{\text{last}}\frac{{{\text{volume}}}}{{{\text{weight}}}}} \right) - \left( {{\text{first}}\frac{{{\text{volume}}}}{{{\text{weight}}}}} \right)}}{{{\text{first}}\frac{{{\text{volume}}}}{{{\text{weight}}}}}} \times 100$$

$${\text{Change in weight percentages of rats}} = \frac{{\text{last weight - first weight}}}{{\text{first weight}}} \times 100$$

## Statistical Analysis

Data were analyzed using IBM SPSS Program version 21.0 (IBM Corp. Released 2012. IBM SPSS Statistics for Windows, Version 21.0. New York, USA). The conformity of the data to the normal distribution was evaluated using the Shapiro–Wilk test and Q–Q graphics, while the Levene test was used to evaluate variance homogeneity. One-way analysis of variance (ANOVA) was used for intergroup comparisons meeting parametric assumptions. Welch’s ANOVA was applied for non-normally distributed data. Tukey HSD test was used for multiple group comparisons. Crosstabs and Chi-square tests were used for qualitative variable comparisons. Statistical significance was set at *p* < 0.05 in all tests.

## Results

### Macroscopic Findings

Two rats died due to anesthesia complications and were excluded from the study. No complications, such as infection, abscess, hematoma, or seroma, were observed. The overall results for the rats and grafts are listed in Table 1.

**Table 1:** Overall results of the rats and grafts

	n	Minimum	Maximum	Mean	Std. Deviation
Preop rat weight (g)	40	190.20	464.00	256.9611	54.40071
Preop fat graft volume (cc)	40	.21	.80	.5378	.13076
Preop fat graft weight (g)	40	.30	.81	.5025	.14528
Postop rat weight (g)	40	298.68	485.20	359.2142	47.28509
Postop fat graft volume (cc)	40	.12	.45	.2864	.07522
Postop fat graft weight (g)	40	.12	.41	.2431	.06391
Cysts	40	.00	4.00	1.9167	1.31747
Fibrosis	40	1.00	4.00	2.0833	.87423
Inflammation	40	1.00	4.00	2.1667	.91026
Perilipin	40	143.00	323.00	234.7500	52.90713
PECAM CD31	40	149.00	302.00	219.6667	42.04827
TUNEL	40	196.00	321.00	274.4000	36.71713
Fat graft survival percentage	40	22.22	100.00	55.8201	17.78145
Fat graft volume/weight change percentage	40	− 57.50	112.50	13.5060	32.93991
Rat weight change percentage	40	4.57	68.92	42.0489	16.36281
Valid n (listwise)	40				

Abbreviations: preop: preoperative, postop: postoperative

There was no statistically significant difference in the average volume/weight change percentages between the groups (*p* = 0.614). Similarly, no statistically significant difference was observed in the average fat graft survival percentages between the groups (*p* = 0.451). There was also no statistically significant difference in the weight percentages of the groups (*p* = 0.311) (Table 2).

**Table 2:** Comparative results of variables

	Sum of squares	df	Mean square		F	Sig.
Preop rat weight	Between groups	27593.534	6	4598.922	1.755	.144
	Within groups	75986.762	29	2620.233		
	Total	103580.296	35			
Preop fat graft volume	Between groups	.041	6	.007	.352	.903
	Within groups	.558	29	.019		
	Total	.598	35			
Preop fat graft weight	Between groups	.053	6	.009	.374	.889
	Within groups	.686	29	.024		
	Total	.739	35			
Postop rat weight	Between groups	9758.815	6	1626.469	.689	.660
	Within groups	68496.984	29	2361.965		
	Total	78255.799	35			
Postop fat graft volume	Between groups	.010	6	.002	.253	.954
	Within groups	.188	29	.006		
	Total	.198	35			
Postop fat graft weight	Between groups	.007	6	.001	.264	.949
	Within groups	.136	29	.005		
	Total	.143	35			
Cysts	Between groups	16.217	6	2.703	1.760	.143
	Within groups	44.533	29	1.536		
	Total	60.750	35			
Fibrosis	Between groups	7.917	6	1.319	2.032	.093
	Within groups	18.833	29	.649		
	Total	26.750	35			
Inflammation	Between groups	8.867	6	1.478	2.129	.080
	Within groups	20.133	29	.694		
	Total	29.000	35			

Perilipin	Between groups	76600.617	6	12766.769	17.325	**.000**
	Within groups	21370.133	29	736.901		
	Total	97970.750	35			
PECAM CD31	Between groups	38484.900	6	6414.150	7.950	**.000**
	Within groups	23397.100	29	806.797		
	Total	61882.000	35			
TUNEL	Between groups	19318.300	6	3219.717	6.648	**.002**
	Within groups	6296.500	13	484.346		
	Total	25614.800	19			
Fat graft survival percentage	Between groups	1879.491	6	313.248	.989	.451
	Within groups	9186.807	29	316.786		
	Total	11066.298	35			
Fat graft volume/weight change percentage	Between groups	5108.412	6	851.402	.751	.614
	Within groups	32867.902	29	1133.376		
	Total	37976.314	35			
Rat weight change percentage	Between groups	1924.960	6	320.827	1.250	.311
	Within groups	7445.991	29	256.758		
	Total	9370.950	35			

Abbreviations: Preop: preoperative, postop: postoperative, df: degrees of freedom, sig: significance

## Histological and Immunohistochemical Findings

**Fig. 2:**
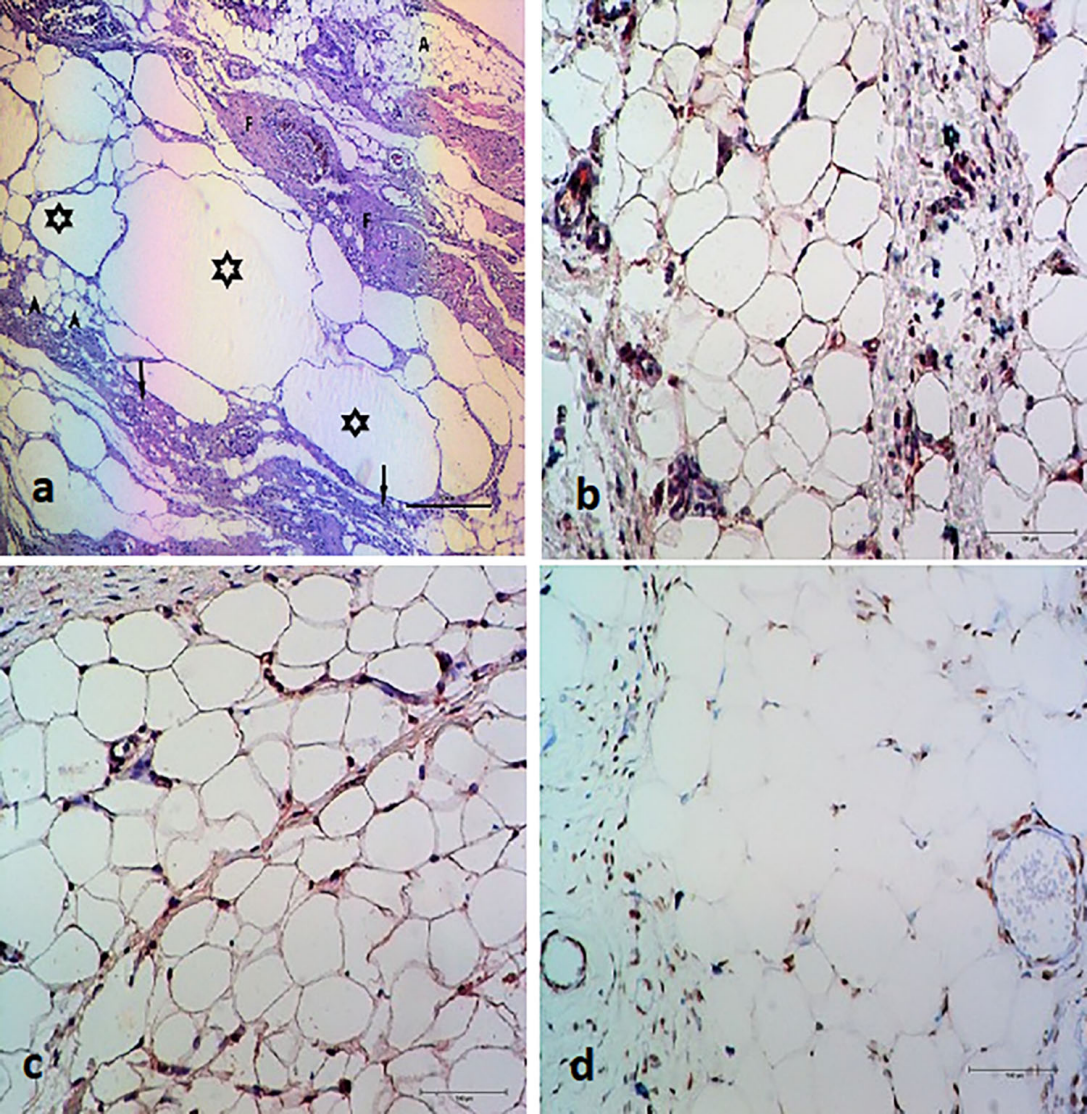
Staining of the excised fat graft,** a**. hematoxylin–eosin,** b**. PECAM,** c**. perilipin,** d**. TUNEL. (asterisk: fat cysts, arrowheads: fat vacuoles, arrows: mononuclear cells, F: areas of fibrosis, A: nucleated live adipocytes)

No statistically significant correlation was found between cyst formation and fibrosis (*p* = 0.361) or between cyst formation and inflammation (*p* = 0.347) across the groups. In addition, there were no statistically significant differences among the groups regarding cyst formation (*p* = 0.199), fibrosis (*p* = 0.070), or inflammation (*p* = 0.318) (Fig. 2, Table 2).

Statistically significant differences were observed between the groups in the average PECAM values (*p* < 0.001). This difference was specifically noted between the following group comparisons: Groups 1 vs. 3 (*p* < 0.001), Groups 1 vs. 4 (*p* < 0.001), Groups 2 vs. 4 (*p* = 0.014), Groups 3 vs. 6 (*p* = 0.022), and Groups 4 vs. 6 (*p* = 0.002). There were also statistically significant differences in the perilipin averages of the groups (*p* < 0.001) (Table 3). This difference was noted in the following group comparisons: Groups 1 vs. 3 (*p* < 0.001), Groups 1 vs. 5 (*p* < 0.001), Groups 1 vs. 7 (*p* < 0.001), Groups 2 vs. 5 (*p* < 0.001), Groups 2 vs. 7 (*p* < 0.001), Groups 3 vs. 6 (*p* < 0.001), Groups 4 vs. 5 (*p* = 0.002), Groups 4 vs. 7 (*p* = 0.001), Groups 5 vs. 6 (*p* < 0.001), and Groups 6 vs. 7 (*p* < 0.001) (Table 3).

**Table 3:** PECAM and perilipin percentages of the groups

PECAM percentages (%)	Group 1	Group 2	Group 3	Group 4	Group 5	Group 6	Group 7	*P* value
n	5	5	6	6	6	6	6	
Mean ± SD	168.0	206.0	252.50	272.40	220.60	192.0	219.60	
	±15.19	±30.53	±38.20	±23.13	±39.29	±17.11	±22.10	<0.001
Min-Max	149.0	168.0	208.0	248.0	171.0	168.0	199.0	
	− 189.0	− 239.0	− 295.0	− 302.0	− 269.0	− 209.0	− 255.0	
Perilipin percentages (%)								
Mean ± SD	178.60	212.80	258.67	217.80	294	178.40	298.20	<0.001
	±20.59	±34.71	±32.53	±28.65	±25.9	±25.89	±16.22	
Min-Max	143.0	159.0	212.0	172.0	255.0	151.0	273.0	
	− 193.0	− 249.0	− 301.0	− 242.0	− 323.0	218.0	316.0	

SD: standard deviation

Lastly, no statistically significant correlation was observed between perilipin and PECAM variables across the groups (r = 0.3202, *p* = 0.057). Similarly, there was no statistically significant correlation between perilipin and TUNEL variables across the groups (r = 0.3853, *p* = 0.093).

## Disussion

Fat grafts are increasingly used in plastic, aesthetic, and reconstructive surgery. Their advantages include low cost, easy accessibility, and biocompatibility. However, they also have major disadvantages, such as varying survival rates and unpredictable postoperative volumes [1, 3].

Several factors affect fat graft viability. The amount of viable fat cells at the time of graft transfer and the vascularity of the donor site significantly impact graft survival. Sezgin et al. preconditioned the fat graft donor areas of rats with microneedling, which increased tissue perfusion. The authors demonstrated longer survival of fat grafts in areas preconditioned by microneedling [4]. In light of this study, we utilized TERUS as a preconditioning method to increase the fat graft survival, as TERUS enhances tissue vascularity. While TERUS did increase vascularity in our study, fat graft survival was decreased in Groups 4 and 6. We attributed this outcome to the fat melting effect of TERUS, consistent with findings from the study conducted by Zhou et al. [18].

An ischemic flap model in rats was designed in a study by Yücel et al., which showed that TERUS reduced flap necrosis by increasing vascularity [9]. Our study investigated fat graft survival in rats preconditioned with TERUS. Based on data gathered, a correlation was identified between increased PECAM values in Groups 2, 4, and 6 and enhanced vascularity due to TERUS. This vascularity increase aligns with findings in the existing literature [19].

TERUS has been shown to reduce fat tissue and tighten skin by stimulating collagen synthesis when used at variable intensities. Both its thermal and non-thermal effects contribute to fat tissue reduction. The thermal effect of TERUS decreases fat tissue through coagulation necrosis [11–13]. Previous studies have demonstrated fat tissue reduction with TERUS-like ultrasonographic devices [12]. In our study, despite the increase in vascularity indicated by PECAM values, a significant increase in live fat tissue was not observed. Our findings on fat tissue are consistent with the literature [18].

Both massage groups and TERUS groups were shown to have increased vascularity in a study by Noble et al. [20]. In our study, the vascularity increase observed in TERUS device-off groups was attributed to the massage effect. In addition, an increase in perilipin levels in TERUS device-off groups was associated with increased vascularity. However, Park et al. reported that endothelial progenitor cells migrated to ischemic tissue and initiated neovascularization, forming vascular cords by the 14th day and fully functional vessels by the 21st day [21]. In our study, although an increase in vascularity was observed histopathologically, a decrease in fat graft viability was determined. This reduction may be due to the non-functionality of the new vessels and the use and duration of TERUS.

## Conclusion

In this study, we investigated the effect of TERUS on fat graft survival. TERUS application was shown to increase vascularity; however, it also reduced fat graft volume, negatively affecting fat graft survival. We consider that the decrease in fat graft survival in the group with postoperative TERUS was due to the diminishing effect of this application on fat tissue. Preconditioning with TERUS can be used to enhance vascularity in fat graft operations, but its postoperative use decreases fat graft survival. We also observed that fat graft survival improved with massage application, as significant differences were observed between the control and massage groups. Further studies on TERUS and fat grafts are necessary, particularly with larger sample sizes.

## References

[1] Simonacci F, Bertozzi N, Grieco MP, Grignaffini E, Raposio E. Procedure, applications, and outcomes of autologous fat grafting. Ann Med Surg. 2017;20:49–60. 10.1016/j.amsu.2017.06.059. 10.1016/j.amsu.2017.06.059 PMC5491488 28702187

[2] Mazzola RF, Mazzola IC. History of fat grafting. Clinics in Plastic Surgery. 2015;42(2):147–53. 10.1016/j.cps.2014.12.002. 25827559 10.1016/j.cps.2014.12.002

[3] Nakamura S, Ishihara M, Takikawa M, et al. Platelet-rich plasma (PRP) promotes survival of fat-grafts in rats. Ann Plast Surg. 2010;65(1):101–6. 10.1097/SAP.0b013e3181b0273c. 20548232 10.1097/SAP.0b013e3181b0273c

[4] Sezgin B, Ozmen S, Bulam H, et al. Improving fat graft survival through preconditioning of the recipient site with microneedling. J Plast Reconstr Aesthet Surg. 2014;67(5):712–20. 10.1016/j.bjps.2014.01.019. 24529693 10.1016/j.bjps.2014.01.019

[5] Hu Y, Jiang Y, Wang M, Tian W, Wang H. Concentrated growth factor enhanced fat graft survival: a comparative study. Dermatol Surg. 2018;44(7):976–84. 10.1097/DSS.0000000000001337. 29894435 10.1097/DSS.0000000000001337

[6] Baek RM, Park SO, Jeong EC, et al. The effect of botulinum toxin A on fat graft survival. Aesthetic Plast Surg. 2012;36(3):680–6. 10.1007/s00266-011-9864-z. 22358314 10.1007/s00266-011-9864-z

[7] Unverdi OF, Coruh A. Effects of microneedle length and duration of preconditioning on random pattern skin flaps in rats. J Plast Reconstr Aesthet Surg. 2020;73(9):1758–67. 32473851 10.1016/j.bjps.2020.03.022

[8] Gersch RP, Fourman MS, Dracea C, Bui DT, Dagum AB. The delay phenomenon: is one surgical delay technique superior? Plast Reconstr Surg Glob Open. 2017;5(10): e1519. 10.1097/GOX.0000000000001519. 29184734 10.1097/GOX.0000000000001519 PMC5682170

[9] Yücel S, Günay GK, Unverdi OF. Effects of ultrasound-assisted preconditioning on critically ischemic skin flaps: an experimental study. Ultrasound Med Biol. 2020;46(3):660–6. 10.1016/j.ultrasmedbio.2019.12.009. 31924418 10.1016/j.ultrasmedbio.2019.12.009

[10] Baker KG, Robertson VJ, Duck FA. A review of therapeutic ultrasound: biophysical effects. Phys Ther. 2001;81(7):1351–8. 11444998

[11] Jewell ML, Solish NJ, Desilets CS. Noninvasive body sculpting technologies with an emphasis on high-intensity focused ultrasound. Aesthetic Plast Surg. 2011;35(5):901–12. 10.1007/s00266-011-9700-5. 21461627 10.1007/s00266-011-9700-5

[12] Mazzoni D, Lin MJ, Dubin DP, Khorasani H. Review of noninvasive body contouring devices for fat reduction, skin tightening and muscle definition. Australas J Dermatol. 2019;60(4):278–83. 10.1111/ajd.13090. 31168833 10.1111/ajd.13090

[13] Sklar LR, El Tal AK, Kerwin LY. Use of transcutaneous ultrasound for lipolysis and skin tightening: a review. Aesthetic Plast Surg. 2014;38(2):429–41. 10.1007/s00266-014-0286-6. 24567045 10.1007/s00266-014-0286-6

[14] Cunha AD, Parizotto NA, Vidal BC. The effect of therapeutic ultrasound on repair of the achilles tendon (tendo calcaneus) of the rat. Ultrasound Med Biol. 2001;27(12):1691–6. 10.1016/S0301-5629(01)00477-X. 11839414 10.1016/s0301-5629(01)00477-x

[15] Young SR, Dyson M. Effect of therapeutic ultrasound on the healing of full-thickness excised skin lesions. Ultrasonics. 1990;28(3):175–80. 10.1016/0041-624X(90)90082-Y. 2339476 10.1016/0041-624x(90)90082-y

[16] Temiz G, Sirinoglu H, Yesiloglu N, Filinte D, Kaçmaz C. Effects of deferoxamine on fat graft survival. Facial Plast Surg. 2016;32(04):438–43. 10.1055/s-0036-1584236. 27494589 10.1055/s-0036-1584236

[17] Sahin Z, Acar N, Ozbey O, Ustunel I, Demir R. Distribution of Notch family proteins in intrauterine growth restriction and hypertension complicated human term placentas. Acta Histochem. 2011;113(3):270–6. 10.1016/j.acthis.2009.10.006. 19913284 10.1016/j.acthis.2009.10.006

[18] Zhou B, Leung BYK, Sun L. The effects of low-intensity ultrasound on fat reduction of rat model. BioMed Research International. 2017;2017:1–8. 10.1155/2017/4701481. 10.1155/2017/4701481 PMC5587957 28913353

[19] Young SR, Dyson M. The effect of therapeutic ultrasound on angiogenesis. Ultrasound Med Biol. 1990;16(3):261–9. 10.1016/0301-5629(90)90005-w. 1694604 10.1016/0301-5629(90)90005-w

[20] Noble GJ, Lee V, Griffith-Noble F. Therapeutic ultrasound: The effects upon cutaneous blood flow in humans. Ultrasound Med Biol. 2007;33(2):279–85. 10.1016/j.ultrasmedbio.2006.08.001. 17306698 10.1016/j.ultrasmedbio.2006.08.001

[21] Park S, Tepper OM, Galiano RD, Capla JM, Baharestani S, Kleinman ME, et al. Selective recruitment of endothelial progenitor cells to ischemic tissues with increased neovascularization. Plast Reconstr Surg. 2004;113(1):284–93. 14707648 10.1097/01.PRS.0000091169.51035.A5

